# Epidemiological Characteristics of Inpatients Undergoing Surgery for Glaucoma at Tianjin Eye Hospital from 2013 to 2017

**DOI:** 10.1155/2021/3628481

**Published:** 2021-11-08

**Authors:** Jin Yang, Yue Qu, Boai Li, Zelong Zhong, Liukun Shi, Xiaofeng Tian, Rui Wang, Dan Xu, Yufeng Liu

**Affiliations:** ^1^Clinical College of Ophthalmology of Tianjin Medical University, Tianjin Eye Hospital, Tianjin Key Laboratory of Ophthalmology and Visual Science, Tianjin Eye Institute, Tianjin 300020, China; ^2^Tangshan Eye Hospital, Tangshan, Hebei 063000, China; ^3^Dazhou Center Hospital, Dazhou, Sichuan 635099, China

## Abstract

**Purpose:**

To analyze the epidemiological characteristics of inpatients who underwent surgery for glaucoma at Tianjin Eye Hospital from 2013 to 2017.

**Methods:**

All glaucoma inpatients who underwent surgery at Tianjin Eye Hospital from 2013 to 2017 were evaluated. The relationships of age and sex with different types of glaucoma were analyzed. The differences in the prevalence and family history of glaucoma among patients with different systemic diseases were compared. Additionally, the effects of different surgical methods for primary angle-closure glaucoma (PACG) and primary open-angle glaucoma (POAG) were compared.

**Results:**

A total of 4539 patients with glaucoma were retrospectively analyzed. The most prevalent type was PACG (60.15%), followed by secondary glaucoma (SG, 25.53%), POAG (7.6%), uncontrollable intraocular pressure (IOP) after antiglaucoma surgery (4.71%), mixed glaucoma (MG, 10%), and congenital glaucoma (CG, 0.9%). The main surgical methods were phacoemulsification (phaco), phacotrabeculectomy (phaco-trab), and trabeculectomy (trab). The rate of phaco-trab increased, while that of trab decreased. The proportion of women in the PACG group was higher than those in the POAG and SG groups, and there was a pronounced tendency for family clustering (*P* < 0.001), while in the POAG and SG groups, the proportions of men and those with diabetes were higher (*P* < 0.05).

**Conclusions:**

In Tianjin Eye Hospital from 2013 to 2017, the main type of glaucoma was PACG. Female sex and a family history of glaucoma were risk factors for PACG, while male sex and hyperglycemia were risk factors for POAG and SG. Among the antiglaucoma surgery methods, the proportion of phaco-trab increased, while the proportion of trab decreased.

## 1. Introduction

Glaucoma is an irreversible eye disease that causes blindness worldwide. It is estimated that, by 2040, there will be 111.8 million cases of glaucoma among those who are 40 to 80 years old [1]. Epidemiological data have indicated that glaucoma incidence varies by type, region, race, sex, and age. In 2011, the prevalence of primary angle-closure glaucoma (PACG) in Harbin County, northeast China, was 1.57%, and the prevalence of primary open-angle glaucoma (POAG) was 0.71% [2]. In the same year, the prevalence of POAG in Pudong New District of Shanghai was 2.8% [3]. A meta-analysis showed that Chinese females were more likely to suffer from PACG than males, with a male-to-female ratio of 1 : 1.75 [4]. Domestic studies have shown that the incidence of glaucoma increases with age [5, 6]. Tianjin has its own unique climate environment and social, economic, and cultural development characteristics. Tianjin Eye Hospital is a tertiary grade A specialized hospital with many inpatients and abundant clinical data. This study retrospectively analyzed the data of inpatients with glaucoma in Tianjin Eye Hospital from 2013 to 2017 and summarized the distributions of glaucoma types, age and sex. The study also analyzed the past disease history of these inpatients and the effects of different antiglaucoma operations on vision and intraocular pressure (IOP) to clarify the development and occurrence trends of glaucoma in Tianjin and provide data support for the prevention and treatment of glaucoma.

## 2. Methods

### 2.1. Subjects

A total of 4539 eyes belonging to 4539 patients who underwent antiglaucoma surgery at Tianjin Eye Hospital from January 1, 2013, to December 31, 2017, were enrolled. Glaucoma was classified into 6 categories according to etiology and the results of gonioscopy: PACG, POAG, secondary glaucoma (SG), mixed glaucoma (MG), congenital glaucoma (CG), and uncontrolled IOP after antiglaucoma surgery. The process of data collection and research followed the Declaration of Helsinki and was approved by the ethics committee of Tianjin Eye Hospital.

### 2.2. Research Methods

The inclusion criteria were as follows: (1) patients with a discharge diagnosis of glaucoma and (2) patients who underwent surgery to treat glaucoma.

The exclusion criteria were as follows: (1) patients who were not treated for glaucoma, (2) patients who were admitted to the hospital repeatedly during the study period (only the first admission data related to glaucoma surgery were retained), or (3) patients with incomplete data.

### 2.3. Ophthalmic Examination

#### 2.3.1. Best-Corrected Visual Acuity

The best-corrected visual acuity (BCVA) was measured with a national standard vision chart.

#### 2.3.2. Intraocular Pressure

IOP was measured with a noncontact tonometer (TX-F, Canon, Japan). If corneal edema or the IOP was too high to be detected, a rebound tonometer (V1-01510, Icare, Finland) was used.

#### 2.3.3. Anterior Structure

A slit-lamp biomicroscope (BM900, Swiss Hager) was used to check the diameter and depth at the center of the cornea; the peripheral anterior chamber; the shape and color of the iris; the shape and diameter of the pupil; light reflection; and the position, thickness, and appearance of the lens. In some cases, the anterior chamber depth was measured by an ultrasound biomicroscope (UBM, MD-300L, Tianjin Mida).

#### 2.3.4. Gonioscopy

The anterior chamber angle was examined with a gonioscope (G-4, VOLK, USA). Scheie classification of the anterior chamber angle was performed.

#### 2.3.5. Fundus

A 90D lens (B104235, VOLK, USA) or ophthalmoscope (YZB, Suzhou Sixty-six) was used to evaluate the cup/disc (C/D), vitreous body, retina, and so forth. Fundus photography was performed in some cases.

#### 2.3.6. Perimetry

Most subjects underwent standard automated perimetry (Octopus, Switzerland, or Humphrey HFA 750i, Zeiss, Germany). Those with PACG in the acute attack stage underwent standard automated perimetry after corneal edema was reduced by topical IOP-lowering medications, intravenous drops of hypertonic agents, or anterior chamber puncture. Uncooperative patients did not undergo the examination.

### 2.4. Statistical Analysis

All data were entered into a Microsoft Excel spreadsheet and proofread by 2 authors. Statistical analyses were performed with SPSS ver.19.0 statistical software. Measurement data are expressed as the means ± standard deviation ($\bar{x}$ ± *s*) or medians (quartiles), and count data are expressed as frequencies. The chi-square test was used to compare count data, while logistic regression was used to assess the relationship between different types of glaucoma and sex, systemic disease, and family history of glaucoma. Pearson correlation analysis was used to analyze the annual variation trend of the average age of glaucoma patients. VA and IOP were compared before and after glaucoma surgery using paired *t*-tests or paired rank-sum tests. A *P* value <0.05 was considered statistically significant.

## 3. Results

### 3.1. Analysis of All the Clinical Characteristics and Demographic Data of Inpatients with Glaucoma

**Figure 1:**
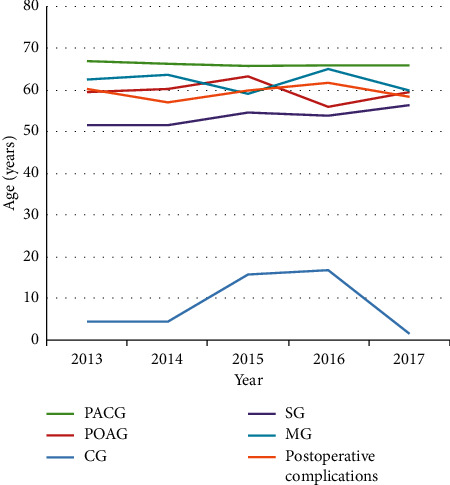
The trend of average age of glaucoma patients from 2013 to 2017 (years). PACG = primary angle-closure glaucoma, POAG = primary open-angle glaucoma, SG = secondary glaucoma, MG = mixed glaucoma, and CG = congenital glaucoma.

A total of 4539 hospitalized glaucoma patients were enrolled in this study with an average age of 61.51 ± 14.43 years. Among them, 1947 patients (42.90%) were male, and 2592 patients (57.10%) were female, with a male-female ratio of 1 : 1.33. The average ages of PACG, POAG, CG, SG, and MG patients were 66.07 ± 9.59, 59.36 ± 16.03, 9.94 ± 14.92, 53.72 ± 16.15, and 60.86 ± 13.23 years, respectively. The chi-square test was used to compare differences in sex distributions, and the difference was not statistically significant (*P*=0.67). Pearson correlation analysis was used to analyze annual variation in the average age and indicated that the average age of glaucoma patients changed over time.  Figure 1 shows the changes in the average age of patients. From 2013 to 2017, the average age of all patients changed from 60.90 to 62.25 years (linear correlation coefficient: *r* = 0.039, *P*=0.009). The average age of SG patients increased from 51.58 to 56.34 years (*r* = 0.10, *P*=0.004), while the average age of patients with other types of glaucoma did not significantly change.

### 3.2. Changes in the Types of Glaucoma from 2013 to 2017

Chi-square tests were used to compare differences in the ratios of different types of glaucoma in different years. The distribution of the number of glaucoma cases of each type per year is shown in Table 1. The results indicated that the difference in the glaucoma composition ratio from 2013 to 2017 was statistically significant. There was a large difference in the composition of glaucoma between 2017 and 2013 (*χ*^2^ = 89.74, *P* < 0.001). PACG (60.15%) was the main type, followed by SG (25.53%), POAG (7.6%), postoperative complications (4.71%), MG (1.10%), and CG (0.9%). The proportions of PACG and SG decreased from 59.48% to 58.75% and from 29.09% to 23.29%, respectively, while that of POAG increased from 7.24% to 9.13%.

**Figure 2:**
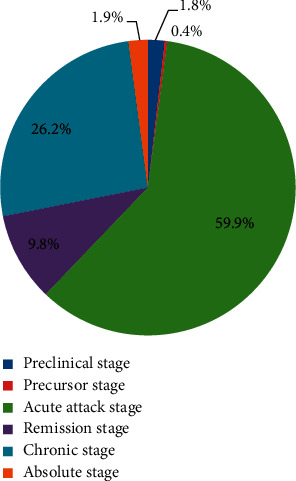
Clinical stages of acute angle-closure glaucoma.

Figure 2 shows the distribution proportion of acute angle-closure glaucoma (AACG) at different clinical stages. The most common primary stage of AACG in hospitalized patients was the acute attack stage (59.93%), followed by the chronic stage (26.17%) and remission stage (9.84%).

**Figure 3:**
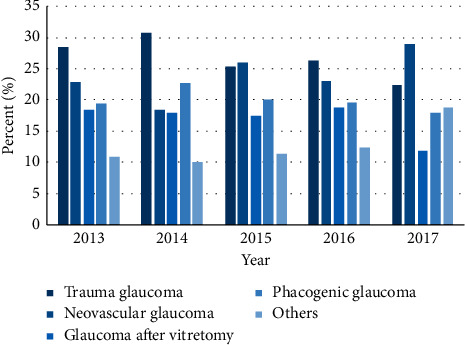
Annual distribution of the different types of secondary glaucoma.

Chi-square tests were used to compare the differences in the ratios of SG subtypes in different years; the results showed that there were no significant differences in the constituent ratios (*χ*^2^ = 24.5, *P*=0.079, Figure 3). The primary types of SG were traumatic glaucoma (26.40%) and neovascular glaucoma (24.16%), followed by phacogenic glaucoma (19.93%), which included the cataract expansion stage and mature stage, hypermature cataracts with cortical liquefaction, and lens subluxation; 16.74% of SG occurred secondary to vitrectomy. Malignant glaucoma, iridocorneal endothelial syndrome, Fuchs syndrome, iridocyclitis, uveitis, exfoliative glaucoma, Axenfeld-Rieger syndrome, endophthalmitis, keratitis, hyperthyroidism, diabetic retinopathy, and complications after cataract surgery accounted for 12.77%.

### 3.3. Association of Sex, Systemic Disease, and Family History among Glaucoma Subgroups

Chi-square tests were used to compare the correlations of single factors (sex, hypertension, diabetes, cerebral infarction, and family history of glaucoma) with PACG, POAG, SG, MG, and postoperative complications (there was no history of chronic disease in the CG group). The results showed that differences in sex, hypertension, diabetes, heart disease, and family history of glaucoma existed among the groups, while no significant difference in cerebral infarction was observed (Table 2). Among all the glaucoma patients, 30.2% had hypertension, 14.4% had diabetes, 11.0% had heart disease, 3.7% had cerebrovascular disease, and 4.2% had a family history of glaucoma. The prevalence of systemic diseases and a history of family glaucoma had different distributions among the various types. The proportion of inpatients with hypertension was highest in the PACG group (33.5%), followed by the POAG group (28.7%), MG group (26.0%), and SG group (25.5%). The prevalence of diabetes was highest in the SG group (20.6%), followed by the POAG group (18.8%), MG group (14.0%), PACG group (11.8%), and postoperative complications group (10.3%). The proportion of heart disease ranked from highest to lowest was in the PACG group (12.7%), POAG group (11.9%), postoperative complications group (8.9%), SG group (7.9%), and MG group (4.0%). The proportion of cerebrovascular disease was highest in the POAG group, accounting for 4.3%, followed by the PACG group (3.9%). The PACG group had the highest proportion of family history of glaucoma (5.8%), followed by the postoperative complications group (5.1%), MG group (4.0%), POAG group (2.9%), CG group (2.4%), and SG group (0.6%). The results showed that glaucoma types were related to sex, diabetes, family history of glaucoma, hypertension, and heart disease (*χ*^2^ = 28.7 to 468.9, *P* < 0.001). The correlation coefficients, from highest to lowest, were 0.321 for sex, 0.121 for diabetes, 0.113 for family history, 0.106 for hypertension, and 0.079 for heart disease, while a history of cerebrovascular disease was not related to any type of glaucoma (*χ*^2^ = 5.6, *P*=0.346).

### 3.4. Types of Antiglaucoma Surgery from 2013 to 2017

Antiglaucoma surgery included trabeculectomy (trab), phacotrabeculectomy (phaco-trab), phacoemulsification (phaco), laser peripheral iridectomy (LPI), transscleral diode laser cyclophotocoagulation (TSCP), and glaucoma drainage implant (GDI). The main surgical method in PACG and POAG patients was phaco-trab, accounting for 41.9% and 59.4%, respectively (Table 3). The second most frequent surgical method was trab, accounting for 29.5% and 32.8%, respectively. The number of antiglaucoma surgeries showed a rising trend from 461 cases in 2013 to 714 cases in 2017. Chi-square tests were used to compare the distribution of surgical methods for PACG in different years, and the difference was statistically significant (*χ*^2^ = 125.9, *P* < 0.001). From 2013 to 2017, the ratio of phaco-trab in PACG patients increased from 31.4% to 52.4%, and the ratio of trab decreased from 41.6% to 17.2%.

### 3.5. Intraocular Pressure Difference after Surgery for Various Types of Glaucoma

The mean preoperative IOP, visual field mean deviation (MD), and pattern standard deviation (PSD) of the 4539 patients were 36.06 ± 17.16 mmHg, 15.11 ± 8.51, and 5.56 ± 4.07, respectively.  Table 4 shows the changes in IOP after different surgical methods. Single-sample Kolmogorov-Smirnov tests for preoperative and postoperative IOP showed that IOP had a skewed distribution except in the MG and postoperative complication groups. Therefore, medians (M) and means ± standard deviation were used to describe changes in IOP. The total median preoperative IOP was 34.3 mmHg, the total median postoperative IOP was 14.0 mmHg, and the median IOP difference value was 18.0 mmHg. Rank-sum tests were used to compare preoperative and postoperative IOP in patients with various types of glaucoma. The results showed that all the differences between preoperative and postoperative IOP were significant (*χ*^2^ = 167.9, *P* < 0.001; *χ*^2^ = 136.2, *P* < 0.001). Wilcoxon signed-rank tests were used to compare changes in IOP inpatients undergoing different surgical methods. The results showed that the difference between preoperative IOP and postoperative IOP was significant in the different surgical methods (*Z* = −5.6, −6.1, −12.0, −14.2, −27.9, and −41.8; *P* < 0.001).

### 3.6. Comparison of VA before and after Primary Glaucoma Surgery

Table 5 shows the changes inVA after different surgical methods. Paired rank and inspection tests were used to compare the differences in VA in patients undergoing different surgical methods for primary glaucoma. The results showed that there were large differences in VA after LPI, TSCP, phaco, trab, and phaco-trab in the PACG group (*Z* = −5.7, −2.1, −11.5, −11.5, and −10.3; *P* < 0.05). There was also a difference in VA after phaco-trab in the POAG group (*Z* = −6.7, *P* < 0.001), while there was no statistically significant improvement in VA after trab, TSCP, or GDI (*Z* = −1.9, −0.6, and −0.4; *P* > 0.05).

## 4. Discussion

### 4.1. Distribution of Age among the Various Types of Glaucoma

The median age of glaucoma inpatients was 63 years, and the median age of the PACG group was the highest, consistent with previous reports [7, 8]. The mechanism may be that as age increases, the lens ages and thickens, and the center shifts forward [9]. As a result, the anterior chamber becomes shallow, squeezing and pushing the iris forward and narrowing or even closing the angle of the chamber, leading to an acute attack of glaucoma [10]. The median age in the POAG group was 62 years. An early study discovered that, among those aged 23 to 80 years, the trabecular meshwork cell density in POAG patients was lower than that in normal people, and the density decreased with age [11]. More studies are needed to explore the detailed mechanism between aging and trabecular meshwork cells. The prevalence of POAG and PACG increased with age, and PACG developed faster than POAG [12, 13].

### 4.2. Distribution of Glaucoma Types from 2013 to 2017

With the aging of the population, the number of hospitalized glaucoma patients has increased [12]. PACG is more common in Asians than in Africans or Europeans [1]. This study showed that PACG and SG were the most important types of glaucoma inpatients at Tianjin Eye Hospital, and the findings were similar to those in Shanghai, Jinan, and Beijing [7, 8, 14]. In this study, PACG occurred most frequently, which might be due to anatomical factors of the Chinese population. Studies have indicated that a small eye size, large lens size, thicker peripheral iris and shallow anterior chamber depth, and anterior positioning of ciliary processes are predisposing factors for PACG [11, 15]. A study confirmed that COL11A1 and PLEKHA7 were associated with an increased risk of PACG in the Han Chinese population [4]. According to the results of China's sixth population census, the Han population in China accounts for 91.51% of the total population, with the densest distribution in eastern China. Tianjin is located on the east coast of China, and the Han population accounts for 97.36% of the total population of Tianjin. These factors may have affected the proportions of PACG and POAG in this study.

Our work showed that POAG accounted for 7.6% of the total cases (POAG/PACG = 0.13), which was consistent with the data from Shanghai and Jinan [7, 8]. A meta-analysis of the epidemiology of the general population showed that, in 2015, 54.42% of all glaucoma patients in China had PACG and 39.79% had POAG [12]. However, the ratios of POAG and PACG in our study were low. One reason may be the insidious onset of POAG, and the other may be the popularity of prostaglandin derivatives, which have long-lasting efficacy and safety [16]. It has been proven that prostaglandin derivatives can effectively reduce IOP by 25∼33% [17].

From 2013 to 2017, the proportion of patients in the acute attack stage of AACG declined proportionately, which may be due to the development of glaucoma education. A regional study noted that a higher level of education was associated with lower prevalence of angle-closure glaucoma [18]. In addition, the rate of cataract surgery has rapidly increased recently [19], which reduces the proportion of patients with acute attack of AACG.

### 4.3. Distributions of Sex, Previous Medical History, and Glaucoma Family History

The male-to-female ratio in the PACG group was 1 : 2.26, which was consistent with a meta-analysis of 12 domestic population-based studies [19]. Earlier studies reported that the anterior chamber was shallower, and the anterior chamber angle was narrower in females than in males [20], which suggests that female sex might be a risk factor for PACG. In addition, the prevalence of hypertension in the PACG group in our study (33.5%) was higher than that in the other glaucoma groups. As mentioned above, PACG patients had high prevalence of family history, and several abnormal genes were confirmed to be related to PACG [21, 22].

The male-to-female ratio in the POAG group was 1 : 0.51, which was consistent with research based on hospital [8] and general populations [19]. The incidence of POAG in females was lower than that in males. The possible reason may be that estrogen in females can inhibit ganglion cell apoptosis and reduce the loss of retinal ganglion cells and nerve fibers [23]; moreover, the testosterone metabolism pathway in males is associated with POAG [24]. Another study found that the MT-CO1 V83I gene mutation has a significant correlation with the occurrence of POAG in males, but a correlation was not shown in females [25]. A survey [26] in 2010 indicated that the prevalence of diabetes in China was estimated to be 11.6%. A previous analysis indicated that T2DM influenced the risk for POAG depending on the genotypes at rs25487 [27]. The proportion of diabetes in POAG patients in this study reached 18.84%, showing that POAG occurrence is associated with diabetes. Although the rate of hypertension in POAG patients was high (28.7%), no correlation was shown in the logistic regression analysis.

The difference in the sex distribution in the CG group (male : female = 1 : 0.86) might be genetically related, but this has not been confirmed [28]. The reason for the sex difference in the SG group (male : female = 1 : 0.55) might be due to traumatic glaucoma, which is related to male violence. NVG, as the major type of SG, is mainly caused by diabetic retinopathy [29]. Indeed, the proportion of diabetes in the SG group was higher in our study. Hyperglycemia and retinal ischemia lead to the production of angiogenic factors, including vascular endothelial growth factor (VEGF), basic fibroblast growth factor (bFGF), platelet-derived growth factor (PDGF), insulin-like growth factor-1, and interferon-*α* [30]. With the spread of these angiogenic factors to the anterior segment, the progress of neovascularization accelerates, even in the iris and trabecular meshwork. Another reason for the higher prevalence of diabetes in the SG group could be glaucoma following vitreoretinal surgery for diabetic retinopathy. A previous study noted out that the incidence of secondary high IOP after vitreoretinal surgery was 20.0∼30.6% [31].

### 4.4. Distribution of Antiglaucoma Surgery Types from 2013 to 2017

The main surgical method for PACG and POAG was phaco-trab, followed by trab, in Tianjin Eye Hospital. From 2013 to 2017, the proportion of phaco-trab procedures increased, while the proportion of trab procedures decreased. This result was consistent with those of research in Wenzhou [32] and South Korea [33]. A meta-analysis showed that phaco-trab can decrease IOP and deep anterior chamber depth more than trab. The primary complications in trab were low IOP, a shallow anterior chamber depth, choroidal detachment, and hyphema, while the primary complications of phaco-trab were shallow anterior chamber depth, corneal bedewing, Descemet's membrane wrinkle, and hyphema [34]. The majority of glaucoma patients were middle-aged or elderly and with cataracts. Trab can accelerate lens degeneration, and cataract surgery after trab can increase the failure rate of trab. Therefore, phaco-trab is a better choice for glaucoma patients with lens opacity [35]. In this study, 205 POAG patients who underwent phaco-trab had a median IOP of 22 mmHg, with a mean IOP of 23.81 ± 8.80 mmHg. The average age of these 205 POAG patients was 67.16 ± 9.62 years. Most of them were diagnosed with cataract at the same time, and they were treated with 2 or 3 IOP-lowering medications on average before surgery, but they still had progressive optic nerve injury for which they were treated with phaco-trab.

### 4.5. Distributions of VA and IOP after Surgery

Reducing IOP is an effective method to control the progression of visual field damage [36]. The postoperative IOP was reduced in patients with various types of glaucoma, indicating that surgery can effectively control the progression of glaucoma. The overall blinding rate of PACG in our study was 33.4%. A meta-analysis of Chinese populations showed that, after removing heterogeneous sources, the total blinding rate of PACG in some areas without popularized prevention was 35.3%. Glaucoma is still a major disease causing blindness in children and adolescents [37]. The blinding rate of CG in this study was 70.7%, and the postoperative VA improvement was not statistically significant, indicating that glaucoma is very harmful to children. Postoperative VA improved in PACG, POAG, and SG patients, indicating that timely surgical intervention was beneficial to improve the prognosis in some glaucoma patients [26].

The limitation of this study is the selection of inpatients for research, resulting in potential selection bias. The epidemiology of glaucoma in the general Chinese population cannot be estimated. Further investigations of the epidemiology of glaucoma in general patients need to be carried out.

## 5. Conclusions

PACG was the most common type of glaucoma in Tianjin Eye Hospital. The acute attack stage is still the most common clinical stage of acute PACG. Traumatic SG was the most common type of SG, followed by neovascular glaucoma.

CG was mostly observed in children, while the other glaucoma groups mostly comprised elderly patients. The proportions of females, glaucoma family history, and hypertension were higher in PACG. POAG and SG were more common in males than in females, and these patients had a higher rate of diabetes.

From 2013 to 2017, the proportion of phaco-trab procedures for primary glaucoma increased, while the proportion of trab procedures decreased. IOP decreased significantly in all types of glaucoma. The improvement in postoperative VA in PACG, POAG, and SG was statistically significant.

## Figures and Tables

**Table 1:** Distribution of glaucoma types from 2013 to 2017.

Year	PACG	POAG	CG	SG	MG	Postoperative complications	Total
2013	411 (59.5%)	50 (7.2%)	9 (1.3%)	201 (29.1%)	4 (0.6%)	16 (2.3%)	691 (100.0%)
2014	421 (56.2%)	56 (7.5%)	8 (1.1%)	211 (28.2%)	12 (1.6%)	41 (5.5%)	749 (100.0%)
2015	613 (59.1%)	56 (5.4%)	10 (1.0%)	293 (28.3%)	20 (1.9%)	45 (4.3%)	1037 (100.0%)
2016	667 (66.0%)	87 (8.6%)	10 (1.0%)	209 (20.7%)	2 (0.2%)	35 (3.5%)	1010 (100.0%)
2017	618 (58.7%)	96 (9.1%)	4 (0.4%)	245 (23.3%)	12 (1.1%)	77 (7.3%)	1052 (100.0%)
Total	2730 (60.1%)	345 (7.6%)	41 (0.9%)	1159 (25.5%)	50 (1.1%)	214 (4.7%)	4539 (100.0%)

PACG = primary angle-closure glaucoma, POAG = primary open-angle glaucoma, CG = congenital glaucoma, SG = secondary glaucoma, and MG = mixed glaucoma.

**Table 2:** Univariate analysis of different factors in different glaucoma types.

	PACG	POAG	CG	SG	MG	Postoperative complications	Total	*χ* ^2^	*P*
Sex								468.8	<0.001
Male	838	228	22	749	25	85	1947		
Female	1892	117	19	410	25	129	2592		
Hypertension	915	99	0	295	13	48	1370	51.3	<0.001
	33.5%	28.7%	0.0%	25.5%	26.0%	22.4%	30.2%		
Diabetes	322	65	0	239	7	22	655	66.7	<0.001
	11.8%	18.8%	0.0%	20.6%	14.0%	10.3%	14.4%		
Heart disease	347	41	0	91	2	19	500	28.7	<0.001
	12.7%	11.9%	0.0%	7.9%	4.0%	8.9%	11.0%		
Cerebrovascular disease	107	15	0	40	0	5	167	5.6	0.346
	3.9%	4.3%	0.0%	3.5%	0.0%	2.3%	3.7%		
Family history	159	10	1	7	2	11	190	57.6	<0.001
	5.8%	2.9%	2.4%	0.6%	4.0%	5.1%	4.2%		

PACG = primary angle-closure glaucoma, POAG = primary open-angle glaucoma, CG = congenital glaucoma, SG = secondary glaucoma, and MG = mixed glaucoma.

**Table 3:** Distribution of types of antiglaucoma surgery from 2013 to 2017.

	2013	2014	2015	2016	2017	Total
PACG						
Phaco	90 (21.9%)	98 (23.3%)	130 (21.2%)	128 (19.2%)	145 (23.5%)	591 (21.6%)
Phaco-trab	129 (31.4%)	168 (39.9%)	223 (36.4%)	299 (44.8%)	324 (52.4%)	1143 (41.9%)
Trab	171 (41.6%)	120 (28.5%)	203 (33.1%)	204 (30.6%)	106 (17.2%)	804 (29.5%)
LPI	5 (1.2%)	18 (4.3%)	32 (5.2%)	7 (1.0%)	10 (1.6%)	72 (2.6%)
TSCP	16 (3.9%)	17 (4.0%)	25 (4.1%)	29 (4.3%)	33 (5.3%)	120 (4.4%)
POAG						
Phaco-trab	29 (58.0%)	31 (55.4%)	34 (60.7%)	43 (49.4%)	68 (70.8%)	205 (59.4%)
Trab	17 (34.0%)	17 (30.4%)	20 (35.7%)	35 (40.2%)	24 (25.0%)	113 (32.8%)
TSCP	4 (8.0%)	4 (7.1%)	0 (0.0%)	4 (4.6%)	4 (4.2%)	16 (4.6%)
GDI	0 (0.0%)	4 (7.1%)	2 (3.6%)	5 (5.7%)	0 (0.0%)	11 (3.2%)
Total	461	477	669	754	714	3075

PACG = primary angle-closure glaucoma, POAG = primary open-angle glaucoma, Phaco = phacoemulsification, Phaco-trab = phacotrabeculectomy, Trab = trabeculectomy, LPI = laser peripheral iridectomy, TSCP = transscleral diode laser cyclophotocoagulation, and GDI = glaucoma drainage implant.

**Table 4:** Changes in IOP after different surgical methods for primary glaucoma (M, mmHg).

	Preoperative IOP	Postoperative IOP	IOP difference	*Z*	*P*
PACG					
Phaco	18.5	13.0	4.0	−14.5	<0.001
Phaco-trab	32.0	13.0	18.0	−27.8	<0.001
Trab	43.0	13.0	27.0	−23.7	<0.001
LPI	61.0	11.0	49.5	−7.4	<0.001
TSCP	46.1	17.0	29.5	−9.4	<0.001
POAG					
Phaco-trab	22.0	16.0	7.0	−10.2	<0.001
Trab	29.0	14.0	15.0	−8.6	<0.001
TSCP	32.5	18.0	14.5	−3.5	0.001
GDI	33.0	9.0	27.0	−2.9	0.003

IOP = intraocular pressure, PACG = primary angle-closure glaucoma, POAG = primary open-angle glaucoma, Phaco = phacoemulsification, Phaco-trab = phacotrabeculectomy, Trab = trabeculectomy, LPI = laser peripheral iridectomy, TSCP = transscleral diode laser cyclophotocoagulation, and GDI = glaucoma drainage implant.

**Table 5:** Changes in VA after different surgical methods for primary glaucoma.

	Preoperative VA			Postoperative VA			*Z*	*P*
	<0.05	≥0.05 and <0.3	≥0.3	<0.05	≥0.05 and <0.3	≥0.3		
PACG								
Phaco	189	255	147	79	227	285	−11.5	<0.001
Phaco-trab	331	459	353	177	488	478	−10.3	<0.001
Trab	257	213	334	82	307	415	−11.5	<0.001
LPI	32	19	21	6	10	56	−5.7	<0.001
TSCP	104	13	3	97	16	7	−2.1	0.032
POAG								
Phaco-trab	47	101	57	26	64	115	−6.7	<0.001
Trab	17	34	62	11	34	68	−1.9	0.058
TSCP	12	4	0	11	5	0	−0.6	0.564
GDI	2	3	6	2	4	5	−0.4	0.655

VA = visual acuity, PACG = primary angle-closure glaucoma, POAG = primary open-angle glaucoma, Phaco = phacoemulsification, Phaco-trab = phacotrabeculectomy, Trab = trabeculectomy, LPI = laser peripheral iridectomy, TSCP = transscleral diode laser cyclophotocoagulation, and GDI = glaucoma drainage implant.

## Data Availability

The data supporting the findings of this study are available from the corresponding author upon request.
